# Conservative treatment of acute traumatic left renal vein occlusion: Importance of left gonadal vein, case report

**DOI:** 10.1016/j.ijscr.2020.03.016

**Published:** 2020-03-28

**Authors:** Hyung Jun Kwon, Kyoung Hoon Lim

**Affiliations:** a Department of Surgery, Kyungpook National University Chilgok Hospital, School of Medicine, Kyungpook National University, Daegu, South Korea; b Department of Surgery, Kyungpook National University Hospital, School of Medicine, Kyungpook National University, Daegu, South Korea

**Keywords:** Left renal vein, Acute occlusion, Traumatic, Gonadal vein, Collateral

## Abstract

**Introduction:**

Isolated acute traumatic renal vein occlusion is rare. As both kidneys have limited capsular and peripelvic vein collaterals, acute renal vein occlusion could lead to renal infarction. However, the left renal vein has potential collateral pathways through the gonadal vein.

**Presentation of case:**

A 56-year-old woman was transferred to our trauma center after a pedestrian accident. Computed Tomography (CT) with contrast enhancement showed that no delineation of left renal vein with adjacent retroperitoneal hematoma around renal vessels, but left renal venous flow was being drained through left gonadal vein, therefore, left kidney was not congested. Her serum creatinine concentration was normal. We elected to treat her left renal vein occlusion conservatively because of the collateral pathway into the gonadal vein.

**Discussion:**

Collateral pathway of the left renal venous drainage may be well known to urologists or vascular surgeons, but may be unfamiliar to trauma surgeons. Therefore, trauma surgeon’s attempts for revascularization of thrombosed left renal vein may lead to massive bleeding or nephrectomy.

**Conclusion:**

Acute left renal vein occlusion close to the inferior vena cava can result in temporary venous hypertension and congestion followed by complete or nearly complete return of function as collateral veins enlarge. If the gonadal vein is patent, left renal vein occlusion could be treated conservatively.

## Highlights

• A blunt renal injury commonly leads to thrombotic partial renal infarct, but only rarely to an isolated acute renal vein occlusion.
• The surgical approach of thrombosed renal vein for preserving the kidney is likely to be an extensive operation, otherwise nephrectomy.
• Collateral pathway of the left renal venous drainage may be well known to urologists, but may be unfamiliar to trauma surgeons.
• If the gonadal vein is patent, left renal vein occlusion could be treated conservatively.

## 1 Introduction

Trauma-induced renal vein occlusion usually presents in combination with renal arterial or parenchymal injury. Isolated acute traumatic renal vein occlusion is very rare. Although intra-abdominal major vascular occlusion may present difficulties to trauma surgeons who are not vascular specialists, several treatment options are available to trauma surgeons with detailed anatomical knowledge, as in the treatment of left renal vein occlusion. In contrast to the right renal vein, the left renal vein has potential collateral pathways. Few reports, however, have described treatment of left renal vein occlusion using these collateral drainage pathways. This report describes the successful conservative treatment of a patient with acute traumatic left renal vein occlusion. This case report has been reported in line with the SCARE criteria [1].

## 2 Presentation of case

**Fig. 1:**
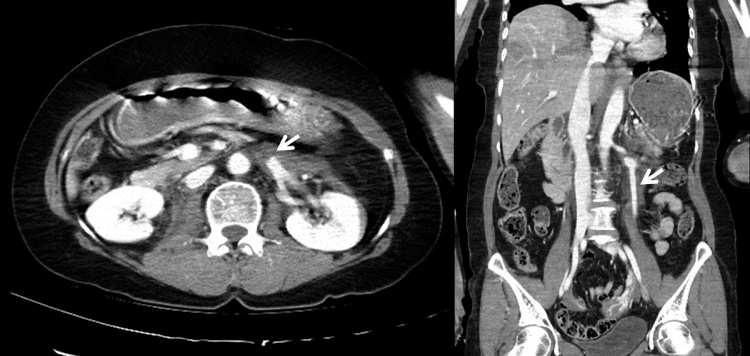
Initial CT scan, showing that the left renal vein was occluded by an adjacent hematoma and the left ovarian vein was patent.

**Fig. 2:**
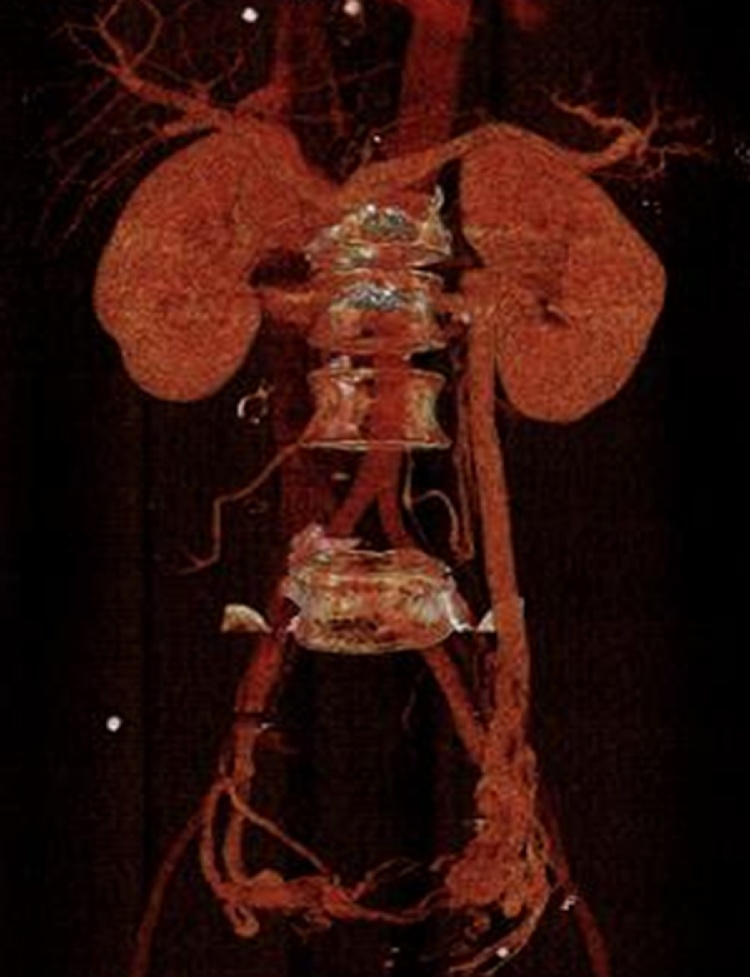
Follow-up CT scan, showing that drainage of the left renal vein was maintained through the ovarian vein.

A 56-year-old woman with no specific medical history was transferred to our trauma center after a pedestrian accident. She presented with alert mentality, mild dyspnea, chest pain, and pelvic pain. Her vital signs were stable and there was no evidence of hypovolemia. Trauma series evaluation revealed multiple left rib fractures, left clavicular fracture, right diaphragmatic rupture, left renal vein occlusion, and pelvic ramus fracture. Abdominal Computed Tomography (CT) with contrast enhancement showed that no delineation of left proximal renal vein with adjacent retroperitoneal hematoma around left renal vessels, but left renal venous flow was being drained through left gonadal vein, therefore, left kidney was not congested (Fig. 1). Her serum creatinine concentration was normal. We elected to treat her left renal vein occlusion conservatively because of the collateral pathway into the gonadal vein. Her right diaphragmatic rupture was repaired laparoscopically. Post-operative anticoagulation therapy was not administered because she was at risk of bleeding from the retroperitoneal hematoma. At 3 month follow up, abdominal CT with contrast enhancement showed that drainage of the left renal vein to the ovarian vein was successfully maintained (Fig. 2). Her blood pressure was normotensive, her renal function was normal, and she had no symptoms of nutcracker syndrome, such as hematuria and left flank pain.

## 3 Discussion

Traumatic renovascular injury, which accounts for only 3%–4% of renal injuries from blunt abdominal trauma, frequently occurs in association with significant injury to the abdominal viscera or skeleton. Traumatic renal vein injury usually occurs in combination with arterial or parenchymal injury [2]. The most common renal pedicle injuries are avulsion and laceration of the main artery and/or vein. Isolated traumatic renal vein occlusion is exceedingly rare. A few reports have described patients with isolated renal vein injury and/or thrombosis after blunt trauma [[2], [3], [4]]. Our patient also had no evidence of concomitant arterial or parenchymal injury. Based on the findings of perivenous hematoma and abrupt cut-off sign, renal vein occlusion in this patient was likely caused by extrinsic compression of a retroperitoneal hematoma following venous laceration.

The clinical presentation of renal vein occlusion in adults depends on the rapidity and degree of venous occlusion, as well as the development of collateral veins. Thus, patients may be asymptomatic, have nonspecific symptoms such as nausea or vomiting, or have more specific symptoms such as hematuria or flank pain suggesting renovascular injury [5].

Because traumatic renal vein occlusion is rare, specific treatment guidelines have not been established. The general treatment goals are bleeding control and preservation of renal function. Historically, patients have been treated with vascular repair, nephrectomy, or conservatively, depending on each patient’s clinical status. Although surgeons may prefer a surgical approach, conservative treatment may be as effective. However, successful conservative treatment requires accurate and detailed anatomical knowledge.

Both kidneys have limited capsular and peripelvic/periureteric vein collaterals, but only the left renal vein has potential collateral pathways through the gonadal, inferior adrenal, and lumbar veins. Acute right renal vein occlusion usually causes venous infarction, whereas acute left renal vein occlusion close to the inferior vena cava produces temporary venous hypotension and congestion followed by complete or nearly complete return of function as collateral veins enlarge [3,6]. Our patient experienced left renal vein occlusion on the right side of the gonadal vein, resulting in venous drainage from the left kidney through the enlarged left gonadal vein.

## 4 Conclusion

Although acute right renal vein occlusion usually causes venous infarction, acute left renal vein occlusion close to the inferior vena cava can result in temporary venous hypertension and congestion followed by complete or nearly complete return of function as collateral veins enlarge. If the gonadal vein is patent, left renal vein occlusion could be treated conservatively.

## Declaration of Competing Interest

None.

## Sources of funding

None.

## Ethical approval

None.

Because this was a report of an interesting case, and not a trial or observational research, there was no need for ethical approval.

## Consent

Written informed consent was obtained from the patient for publication of this case report and accompanying image.

## Author contribution

Hyung Jun Kwon and Kyoung Hoon Lim were involved with the case and writing of the manuscript, general management of the patient and revised the manuscript for important intellectual content.

All authors read and approved the final manuscript.

## Registration of research studies

None.

## Guarantor

Hyung Jun Kwon, Kyoung Hoon Lim.

## Provenance and peer review

Not commissioned, externally peer-reviewed.
